# Protein Extraction From *Mycobacterium* spp. for Identification by MALDI‐TOF Mass Spectrometry

**DOI:** 10.1002/cpz1.70425

**Published:** 2026-07-30

**Authors:** Lucas Evangelista Marques, Stephanie Chrystine Balestro Mota, Christoffel Opperman, Robin Mark Warren, Emilyn Costa Conceição, Cristina Viana‐Niero

**Affiliations:** ^1^ Department of Microbiology, Immunology and Parasitology Federal University of São Paulo São Paulo Brazil; ^2^ South African Medical Research Council Centre for Tuberculosis Research, Division of Molecular Biology and Human Genetics, Faculty of Medicine and Health Sciences Stellenbosch University Cape Town South Africa; ^3^ National Health Laboratory Service Green Point TB‐Laboratory Cape Town South Africa; ^4^ Division of Medical Microbiology, Department of Pathology University of Cape Town Cape Town South Africa; ^5^ Centre for Epidemic Response and Innovation (CERI), School for Data Science and Computational Thinking Stellenbosch University Cape Town South Africa

**Keywords:** MALDI‐TOF, mass spectrometry, *Mycobacterium*, protein extraction, sample preparation

## Abstract

The identification of mycobacteria remains challenging due to the high species diversity within the genus and the lack of a molecular method able to identify them in a single assay. Matrix‐assisted laser desorption/ionization time‐of‐flight mass spectrometry (MALDI‐TOF MS) has been used as a rapid and promising alternative for bacterial identification; however, its reliability is highly dependent on effective protein extraction. This article describes a protocol specifically developed for the identification and species‐level discrimination of *Mycobacterium* spp. that integrates thermal inactivation, multiple biomass washes, mechanical disruption with zirconia beads, protein extraction using formic acid and acetonitrile, quality control, and troubleshooting. This procedure is compatible with Bruker MALDI Biotyper systems and is designed to meet biosafety level (BSL)‐2 and BSL‐3 laboratory requirements, ensuring operator safety and preventing aerosol generation. Emphasis is placed on standardizing critical steps, reproducibility, and safe handling of pathogenic strains. This protocol can be applied in both diagnostic and research laboratories, offering a reliable and standardized approach to preparing mycobacterial samples for analysis by MALDI‐TOF MS. © 2026 The Author(s). *Current Protocols* published by Wiley Periodicals LLC.

**Basic Protocol**: Protein extraction from *Mycobacterium* spp. cultured on solid media for identification by MALDI‐TOF mass spectrometry

**Support Protocol**: Preparation and quality control of mycobacterial cultures for MALDI‐TOF MS analysis

## INTRODUCTION

Accurate identification of *Mycobacterium* species is essential for appropriate clinical management, epidemiological surveillance, and infection control. The genus comprises important human pathogens, such as members of the *Mycobacterium tuberculosis* complex (MTBC), as well as >190 non‐tuberculous mycobacteria (NTM), which are considered opportunistic pathogens associated with pulmonary and extrapulmonary infections. Conventional phenotypic identification methods are labor‐intensive, time‐consuming, and not discriminatory enough, whereas molecular approaches, although faster, often require specialized infrastructure and are generally limited to the detection of a restricted number of species (Lopes et al., 2023; Lyamin et al., 2023; WHO, 2024).

Matrix‐assisted laser desorption/ionization time‐of‐flight mass spectrometry (MALDI‐TOF MS) has improved microbial identification by enabling rapid analysis through the comparison of protein spectra with reference databases, allowing the identification of a large number of species in a single assay (Calderaro & Chezzi, 2024; Weiss & Basu, 2025). This technique has been evaluated since 2006 for the identification of mycobacterial species (Pignone et al., 2006). Since then, several studies have proposed improvements to the method, but to date, there is no consensus on the best protein extraction methodology to be employed (Albay et al., 2022; El Khéchine et al., 2011; Huang et al., 2018; López‐Medrano et al., 2025; Lyamin et al., 2023; Machnik et al., 2024; Mather et al., 2014; Saleeb et al., 2011). Martin et al. (2023) compared the MBT‐Mycobacteria kit and the Easy MycoEx protocol, both developed by Bruker Daltonics, underscoring that protein extraction methods remain an area of active optimization for improved mycobacterial identification.

In the context of mycobacteria, MALDI‐TOF MS represents a comprehensive and efficient approach for microbial identification, supported by commercial libraries, such as those provided by Bruker Daltonics, which currently include profiles of >180 species (Bruker Daltonics, 2026).

**Figure 1:**
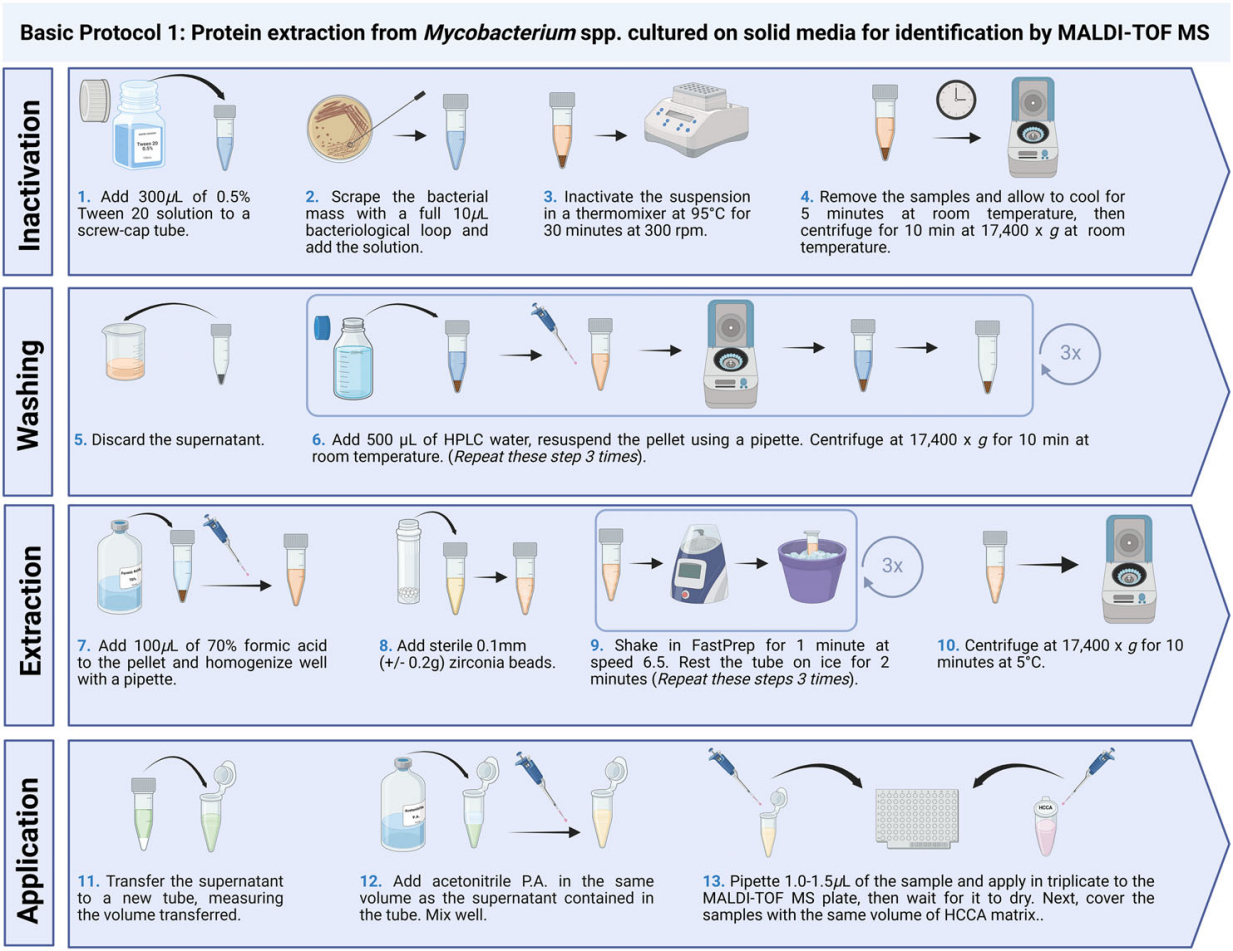
Workflow for identification of *Mycobacterium* species by MALDI‐TOF mass spectrometry created using BioRender.com under a valid license. Schematic overview of the protocol highlighting the main steps: (Inactivation) collection of mycobacterial biomass under biosafety level 2 (BSL‐2) or 3 (BSL‐3) conditions, followed by thermal inactivation in the presence of detergent; (Washing) washing steps to remove residual contaminants and cellular debris; (Extraction) protein extraction by mechanical disruption using zirconia beads combined with formic acid treatment; (Application) application of the protein extract onto the target plate, followed by matrix addition, for species identification using MALDI‐TOF MS.

Considering these limitations, this study proposes a standardized workflow for protein extraction from *Mycobacterium* spp. cultured on solid media for identification by MALDI‐TOF MS. The protocol was developed based on evidence from the literature and experimental optimization, including thermal inactivation, detergent‐assisted washing, mechanical disruption using zirconia beads, and solvent‐based extraction (El Khéchine et al., 2011; Saleeb et al., 2011). The Basic Protocol describes the complete procedure for preparing mycobacterial samples for MALDI‐TOF MS analysis (Figure 1).

## STRATEGIC PLANNING

Successful identification of *Mycobacterium* species by MALDI‐TOF MS depends primarily on the quality of the starting culture and the efficiency of protein extraction. Therefore, users must ensure that isolates are properly processed and cultured. To obtain reliable results, careful attention should be given to critical factors such as culture purity, adequate biomass, and avoidance of culture medium carryover, as these directly impact spectral quality and identification scores.

The Basic Protocol describes a complete workflow for protein extraction from mycobacteria grown on solid media for MALDI‐TOF MS identification. The workflow is summarized in Figure 1, which provides a schematic overview of the key steps involved in sample preparation and analysis.

The Support Protocol outlines the steps before protein extraction, including specimen processing, cultivation, and quality control (QC) procedures. These include confirmation of acid‐fast bacilli (AFB), assessment of colony morphology, and optional antigen‐based screening to differentiate members of the MTBC from NTM.

*CAUTION: Mycobacterium* spp. includes Biosafety Level 2 (BSL‐2) and Biosafety Level 3 (BSL‐3) pathogens. All manipulations involving viable cultures must be conducted according to institutional biosafety regulations.

*CAUTION*: Strains belonging to the MTBC must be handled in BSL‐3 laboratories and manipulated within a certified Class II biological safety cabinet.

*CAUTION*: Preparation and handling of volatile or corrosive chemical reagents (e.g., acetonitrile, trifluoroacetic acid, and formic acid) must be performed in a chemical fume hood, using appropriate personal protective equipment (PPE).

## PROTEIN EXTRACTION FROM *Mycobacterium* spp. CULTURED ON SOLID MEDIA FOR IDENTIFICATION BY MALDI‐TOF MASS SPECTROMETRY

This protocol describes a standardized method for extracting proteins from *Mycobacterium* spp. cultured on solid media for identification by MALDI‐TOF MS. The procedure combines thermal inactivation, washing steps, mechanical disruption using zirconia beads, and protein extraction with an organic solvent. Following these guidelines, this protocol produces high‐quality mass spectra that enable the identification of mycobacterial species.

### Materials

Mycobacterial culture (see Support Protocol)
H_2_O, Milli‐Q
0.5% Tween 20 solution (see recipe)
70% formic acid (see recipe)
Acetonitrile (Thermo Fisher Scientific, cat. no. A998‐1 or equivalent)
HCCA matrix solution (see recipe)
Bacterial Test Standard (BTS) working solution (see recipe)

Class II biological safety cabinet (ESCO Labculture Class II, Type B2 Total Exhaust Biological Safety Cabinet or equivalent)
10‐µl inoculating loops
Screw cap tubes with O‐rings (Kasvi, cat. no. K6‐0151 or equivalent)
Vortex mixer
Thermoblock capable of 95°C incubation (Eppendorf, cat. no. AG 22331 or equivalent)
Microcentrifuge (Eppendorf, cat. no. 5471R or equivalent)
10‐, 50‐, 100‐, and 1000‐µl micropipette tips and pipettes
0.1‐mm zirconia beads (BioSpec Products, cat. no. 11079101z or equivalent)
FastPrep FP120 (Thermo Savant Bio101 or equivalent)
MSP 96 biotarget chip polished steel BC (Bruker, cat. no. 8280800)
Bruker MALDI Biotyper System (Microflex or Sirius)
Bruker MBT Compass flexControl software version 3.4

#### Preparation of cultured isolates for extraction

1 Upon confirmation of a pure mycobacterial culture, transfer ∼1 loopful (∼10 µl) of mycobacterial biomass from a fresh culture into a screw‐cap microcentrifuge tube containing 300 µl of sterile ultrapure water (Milli‐Q) supplemented with 0.5% Tween 20.
  Obtaining adequate biomass is critical for reliable MALDI‐TOF MS identification of both rapidly and slowly growing mycobacteria, as insufficient biomass may compromise spectrum quality and identification scores; therefore, additional subculturing is recommended whenever the initial growth is inadequate for optimal protein extraction.
  Take care when collecting the biomass to avoid transferring fragments of the culture medium into the tube. Egg‐based media, such as Löwenstein–Jensen or Ogawa–Kudoh, may detach together with the biomass.
  To facilitate biomass collection and minimize disruption of egg‐based media, lightly moisten the colony surface using the inoculation loop with a small volume of sterile Milli‐Q water supplemented with 0.5% Tween 20 prior to sampling.
  In addition, gently moving the loop in a horizontal zig‐zag motion along the inner wall of the tube can help release biomass that remains adhered to the culture medium.
  The presence of culture medium can be recognized by a greenish coloration of the suspension.
2 Homogenize the suspension using the inoculating loop or by vortexing for 15 s.

#### Heat inactivation of mycobacterial biomass

3 Incubate the suspension at 95°C in a thermoblock with agitation at 300 rpm for 30 min to ensure inactivation of the mycobacteria.
4 Remove the samples and allow them to cool for 5 min at room temperature.

#### Washing of inactivated biomass

5 Centrifuge the tube 10 min at 17,400 × *g*, room temperature, and carefully discard the supernatant.
6 Add 500 µl of sterile Milli‐Q water to the pellet, homogenize the suspension using a pipette, centrifuge again under the same conditions, and discard the supernatant. Repeat the washing outlined in steps 5 to 6 three times.
  Rinsing is essential to remove residual detergent and the solubilized cellular aggregates generated during the inactivation step.
  Exercise caution when handling rough colony morphotypes, as the pellet may detach from the bottom of the tube during removal of the supernatant. Some biomass loss is expected at this stage, particularly for rough colonies, as cells may adhere to the walls of pipette tips during homogenization.

#### Protein extraction

7 Resuspend the pellet in 100 µl of 70% formic acid.
8 Add ∼0.2 g of sterile 0.1‐mm zirconia beads.
9 Homogenize the sample using a FastPrep FP120 homogenizer for 1 min at speed 6.5.
10 Place the tube on ice and allow it to rest for 2 min.
  Repeat steps 9 and 10 three times.
11 Centrifuge the lysate for 10 min at 17,400 × *g*, 5°C.
12 Transfer the supernatant to a clean microcentrifuge tube and record the volume.
13 Add an equal volume of 100% acetonitrile and mix thoroughly using a pipette.

#### Preparation of the MALDI target plate

14 Apply 1 to 1.5 µl of the extracted protein solution onto each spot of a MALDI target plate, performing at least triplicates.
15 Allow the droplets to air‐dry completely.
16 Overlay each spot with an equal volume of HCCA matrix solution and allow it to dry again.
  The HCCA matrix must be applied to the samples and the bacterial standard (BTS) within 30 min of their drying.
17 Process the plate using a MALDI‐TOF MS instrument, following the manufacturer's instructions.
  The prepared MALDI target plates must be analyzed within 24 hr of preparation.

#### Acquisition of MALDI‐TOF MS spectra

18 Analyze the extracted protein samples using a MALDI‐TOF mass spectrometer, such as the Microflex LT or Sirius system (Bruker Daltonics). Acquire spectra using the FlexControl software v. 3.4 with the MBT HT Mycobacteria Module.
19 Obtain spectra in a mass‐to‐charge (m/z) range of 2000 to 20,000 Da. Measure samples in automatic mode, operating at 40 shots/s, collecting a total of 240 laser shots per spot.

#### Species identification and interpretation of spectra

20 Interpret identification results according to the manufacturer's recommended log(score) thresholds: scores <1.60 indicate unreliable identification, scores between 1.60 and 1.79 indicate low‐confidence identification, and scores ≥1.80 indicate high‐confidence identification.
  A potential limitation of MALDI‐TOF MS–based identification is the analysis of mixed mycobacterial cultures, which may generate composite protein spectra and interfere with accurate species assignment. In these situations, overlapping spectral peaks can result in reduced identification scores, inconsistent replicate results, or preferential identification of the predominant organism.

## PREPARATION AND QUALITY CONTROL OF MYCOBACTERIAL CULTURES FOR MALDI‐TOF MS ANALYSIS

This protocol describes the preparation, cultivation, and quality control (QC) of mycobacterial isolates prior to protein extraction and MALDI‐TOF MS analysis. Proper sample processing and culture conditions are critical for obtaining sufficient, pure biomass, which directly impacts the quality and reproducibility of the resulting mass spectra. In addition, rigorous QC procedures are required to confirm the presence of acid‐fast bacilli and to exclude contamination or mixed cultures, which may compromise species identification. This protocol outlines recommended steps for specimen processing, culture on solid media, and QC procedures, including microscopic examination, optional antigen‐based screening, and macroscopic assessment of colony morphology.

### Materials

Solid media: Middlebrook 7H10 agar with OADC medium (see recipe) or Löwenstein–Jensen medium (LJ; BD Difco, cat. no. 00382902444107), or other suitable solid medium for mycobacterial growth

Class II biological safety cabinet (ESCO Labculture Class II, Type B2 Total Exhaust Biological Safety Cabinet or equivalent)
10‐µl inoculating loops
Incubator, 30° to 37°C

Additional reagents and equipment for: validated digestion, concentration, and/or decontamination appropriate to the sample type (Parish & Kumar, 2021); Ziehl–Neelsen (ZN) staining or auramine‐based fluorochrome staining (Parish & Kumar, 2021); and MPT64 antigen detection testing (e.g., Capilia TB‐Neo or equivalent) (Chikamatsu et al., 2014; Ramos et al., 2016)

#### Clinical sample and mycobacterial culture

1 Before culture, process the specimen using a validated digestion, concentration, and/or decontamination method appropriate to the sample type (Parish & Kumar, 2021).
2 Inoculate the processed sediment onto an appropriate solid medium and incubate at 30° to 37°C until visible colonies develop. This is the primary culture.
  If the primary culture was performed in liquid medium, subculture onto solid medium is recommended to increase mycobacterial biomass before protein extraction and to verify culture purity.
3 After observed growth, perform a QC to verify if the culture is pure and free of contamination before proceeding with protein extraction. Pure cultures are essential to obtain reliable MALDI‐TOF mass spectra.

#### Mycobacterial culture quality control

4 After visible growth is observed, perform culture QC to confirm that the isolate is compatible with downstream protein extraction and MALDI‐TOF MS analysis.
  Pure cultures are essential for generating reproducible spectra and reliable species identification. Culture QC should include microscopic confirmation of acid‐fast bacilli (AFB), visual inspection of colony morphology, and, when required, a rapid antigen‐based screening assay to differentiate MTBC from NTM.
5 Confirm the presence of AFB by Ziehl–Neelsen (ZN) staining or auramine‐based fluorochrome staining (Parish & Kumar, 2021).
  If AFB are observed and no non‐AFB contaminants are seen, proceed to sample preparation for extraction.
  If AFB are observed together with morphologically distinct non‐AFB organisms or obvious mixed background material, do not proceed to extraction. Purify the culture first, either by subculturing an isolated colony onto fresh solid medium or by re‐isolation after serial dilution and then repeat QC before continuing.
6 Optionally perform an MPT64 antigen detection test (e.g., Capilia TB‐Neo or equivalent) on culture material as a rapid screening method to differentiate MTBC from NTM (Chikamatsu et al., 2014; Ramos et al., 2016).
  This step is optional but useful in laboratories processing both MTBC and NTM, particularly when an early distinction is relevant for biosafety handling or workflow triage. A positive result supports classification as MTBC, whereas a negative result is consistent with NTM. However, a negative MPT64 result should not be used as the sole basis for excluding MTBC, because false‐negative MTBC cultures have been reported, including strains carrying mutations affecting the MPT64 target. Therefore, any unexpected negative result should be interpreted cautiously and resolved using the laboratory's confirmatory identification workflow.
  If the MPT64 test is positive, classify the culture as presumptive MTBC and maintain handling under the appropriate biosafety conditions.
  If the MPT64 test is negative, classify the culture as presumptive NTM, while recognizing that additional confirmation may still be required in selected cases.
7 Perform macroscopic inspection of colony morphology before biomass collection to confirm sample purity.
  Inspect the primary culture for evidence of mixed growth, including marked differences in colony size, texture, elevation, pigmentation, opacity, or growth pattern. The presence of more than one distinct colony morphology may indicate contamination or mixed mycobacterial growth. In such cases, do not proceed directly to extraction. Instead, purify the isolate by selecting a single well‐isolated colony for subculture onto fresh solid medium, or by re‐plating after serial dilution, and repeat the QC steps above once new growth is obtained. Visual inspection is particularly important because mixed cultures may still yield misleading spectra even when acid‐fast organisms are present.

## REAGENTS AND SOLUTIONS

### 7H10 agar with OADC medium

Dissolve the appropriate amount of Middlebrook 7H10 agar powder (BD Difco, cat. no. 00382902627104 or equivalent) in deionized water with glycerol (Sigma‐Aldrich, cat. no. G7893 or equivalent), following the manufacturer's instructions. Sterilize the medium by autoclaving (121°C for 15 min). Allow the medium to cool to ∼55°C, then aseptically add Middlebrook OADC enrichment (BD BBL, cat. no. 00382902122401 or equivalent) at the recommended concentration. Mix gently and pour into sterile Petri dishes. Allow the medium to solidify and store up to 1 month at 4°C.

### BTS working solution

Rehydrate lyophilized Bacterial Test Standard (BTS; Bruker, cat. no. 8255343) with 50 µl of organic solvent solution (see recipe) and pipet up and down 20 times, taking care to avoid bubbles or foaming. Repeat pipetting up and down an additional 20 times, taking care to avoid bubbles. Centrifuge for 2 min at 2650 × *g*, room temperature. Aliquot 5 µl into microcentrifuge screw top vials. Label with date of generation and store up to 6 months at −80°C.

You will not recover all 50 µl, as acetonitrile evaporates very rapidly.

### Formic acid, 70% (v/v)

Mix 7 ml formic acid (Sigma‐Aldrich, cat. no. F0507 or equivalent) and 3 ml of HPLC‐grade deionized water to obtain a 70% (v/v) solution. Prepare small volumes for single use (∼10 ml) to reduce degradation and store at room temperature. Prepare the solution on the day of analysis using MS‐grade reagents in a fume hood.

### HCCA matrix solution

Prepare the matrix solution by dissolving α‐cyano‐4‐hydroxycinnamic acid (HCCA; Sigma‐Aldrich cat. no. C8982) in the organic solvent solution (see recipe) to obtain a final concentration of ∼10 mg/ml. For example, dissolve 10 mg HCCA in 1 ml of organic solvent solution. Mix thoroughly using vortex until the matrix is completely dissolved. Store the solution up to 1 month in tightly sealed microcentrifuge tubes at 4°C, protected from light. Before use, allow the solution to reach room temperature and mix again using a vortex to ensure homogeneity.

Precipitation of crystals in the solution during storage is normal. To resuspend, the matrix can be gently heated at 37 °C for 5 min and then vortexed to ensure complete dissolution.

### Organic solvent solution

Prepare a solution containing 50% (v/v) acetonitrile (Thermo Fisher Scientific, cat. no. A998‐1 or equivalent), 47.5% (v/v) HPLC‐grade deionized water, and 2.5% (v/v) trifluoroacetic acid (TFA; Sigma‐Aldrich cat. no. T6508 or equivalent). For example, mix 5 ml acetonitrile, 4.75 ml of deionized water, and 250 µl TFA to obtain 10 ml of organic solvent solution. Mix thoroughly before use. Store the solution up to 6 months in a tightly sealed container at 4°C, protected from light and contamination.

This solution must be prepared in a chemical fume hood while using appropriate personal protective equipment (PPE).

### Tween‐20 solution, 0.5% (v/v)

Prepare a 0.5% (v/v) Tween 20 solution by adding 125 µl Tween 20 (Sigma‐Aldrich cat. no. 9005‐64‐5/P1379 or equivalent) to 24.875 ml of sterile distilled water to obtain a total volume of 25 ml. Mix thoroughly until the solution is homogeneous. Sterilize the solution by autoclaving at 121°C for 15 min or by filtration through a 0.22‐µm sterile filter. Store the sterilized solution up to 3 months in a tightly sealed container at room temperature, protected from light and contamination.

## COMMENTARY

### Background Information

This protocol was developed to provide a reliable approach compatible with biosafety requirements for the extraction of *Mycobacterium* spp. proteins suitable for MALDI‐TOF MS analysis. The workflow takes into account the structural characteristics of mycobacteria, particularly the lipid‐rich cell wall, which can limit protein accessibility when standard bacterial extraction methods are used (Daffé & Marrakchi, 2019; Forbes et al., 2018).

In this context, the proposed workflow incorporates a series of complementary steps designed to overcome the structural barriers of mycobacteria and improve protein extraction efficiency. Thermal inactivation ensures biosafety while also contributing to cell wall destabilization (Wang et al., 2021). The inclusion of Tween 20 during the initial suspension step promotes the dispersion of clumped bacilli and aids in the solubilization of lipid components of the cell wall (El Khéchine et al., 2011; Gordillo‐Marroquín et al., 2022; Julián et al., 2001). Subsequent washing steps are essential to remove residual detergent and solubilized debris that could interfere with spectral acquisition. Mechanical disruption using zirconia beads in the presence of formic acid enhances cell lysis by combining physical shear forces with chemical denaturation, facilitating the release of intracellular proteins (El Khéchine et al., 2011; Saleeb et al., 2011; Toney et al., 2022). Finally, the addition of acetonitrile promotes protein solubilization (Rodriguez‐Temporal et al., 2022; Saleeb et al., 2011; Toney et al., 2022). Together, these steps are designed to address known limitations of conventional protocols.

Currently, the Bruker system provides two main approaches for mycobacterial protein extraction: an in‐house protocol (MycoEx), which can be performed using standard laboratory reagents, and a commercially available kit (Easy MycoEX). To illustrate the differences between these workflows and the protocol proposed in this study, the main steps of each method, including inactivation, washing, extraction, and application, are summarized in Table 1.

**Table 1:** Comparison of Protein Extraction Protocols for *Mycobacterium* spp. for Analysis by MALDI‐TOF MS*^a^*

Protocol	Inactivation	Washing	Extraction	Application
This study	95°C for 30 min in HPLC water and 0.5% Tween 20	Three stages of washing with Milli‐Q water	Resuspended pellet in 70% formic acid and 0.1‐mm beads, Fast Prep agitation three times with cooling on ice between cycles; the supernatant was mixed with an equal volume of 100% acetonitrile	The supernatant was spotted onto the MALDI‐TOF MS target plate and overlaid with HCCA matrix
MycoEx (Alcaide et al., 2018; Bacanelli et al., 2023; Bruker Daltonics, 2014; Martin et al., 2023; Pastrone et al., 2023)*^b^*	95‐100°C for 30‐60 min in HPLC‐grade water	70‐100% ethanol	Resuspended pellet in 100% acetonitrile and 0.5‐mm beads and a vortex mixer; add 70% formic acid, vortex, and centrifuge	The supernatant was applied to the MALDI‐TOF MS target plate and overlaid with HCCA matrix
Easy MycoEx (Bruker Daltonics, 2026; Martin et al., 2023; Pastrone et al., 2023)	Washing solution + Inactivation reagent for 30 min at room temperature		Resuspended pellet in 100% acetonitrile and 70% formic acid, vortex, and centrifuge	The supernatant was applied to the MALDI‐TOF MS target plate and overlaid with HCCA matrix

^*a*^ The table illustrates the main differences between the proposed protocol and the methods recommended by the manufacturer (MycoEx and Easy MycoEX, Bruker Daltonics), highlighting the workflow steps of each procedure.

^*b*^ Minor variations in inactivation temperature (95‐100°C for 30‐60 min) and ethanol concentration (70‐100%) have been reported across studies; however, all protocols follow the same general workflow as the MycoEx method described by Bruker Daltonics (2014).

#### Sample data

The Bruker Daltonics mycobacterial library currently contains reference spectra for mycobacterial species, including members of the MTBC and a broad range of NTM. Identification by MALDI‐TOF MS–based is dependent on the reference database used for spectral matching. Therefore, identification accuracy may be affected by the current coverage of the Bruker Mycobacteria Library, which presently includes 182 of the 201 recognized *Mycobacterium* species (Bruker Daltonics, 2026).

Spectra generated using this protocol are analyzed with MBT Compass HT software (Bruker Daltonics), which compares spectra acquired from each isolate with reference profiles in the selected database and assigns an identification score (ID score) ranging from 0.00 to 3.00 (Bruker Daltonics, 2026).

When the MBT Mycobacteria library is used, ID scores of 0.00 to 1.59 indicate that no reliable identification was obtained, scores of 1.60 to 1.79 indicate low‐confidence identification, and scores of 1.80 to 3.00 indicate high‐confidence identification. In this protocol, scores within the high‐confidence range represent the desired outcome, as they are consistent with reliable species‐level identification (Bruker Daltonics, 2026).

**Figure 2:**
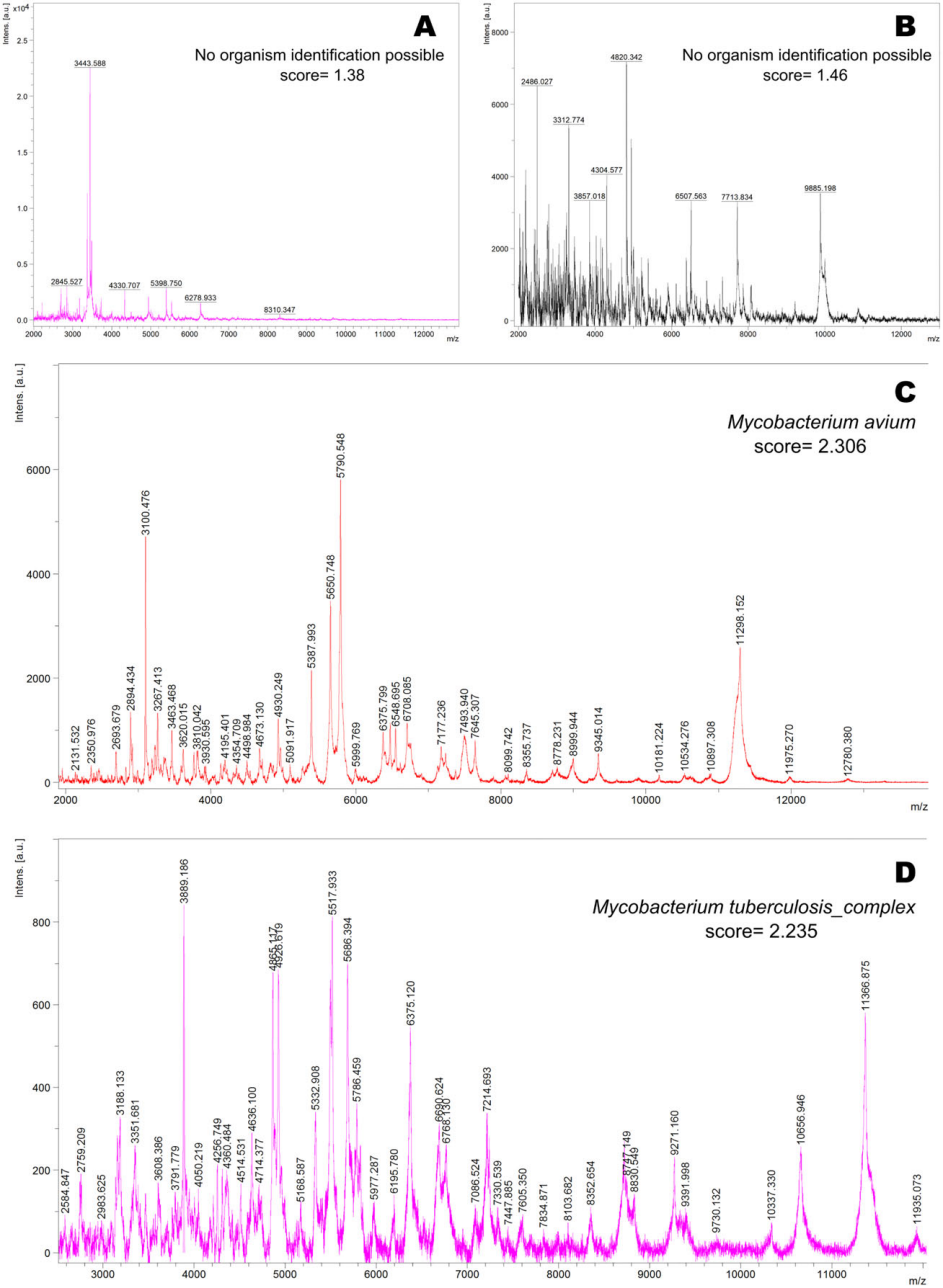
Representative MALDI‐TOF MS spectra illustrating spectrum quality and identification outcomes for mycobacterial isolates. (**A**) Spectrum with insufficient signal intensity resulting from inadequate biomass, leading to failed identification. (**B**) Spectrum showing poor quality due to sample contamination, preventing reliable identification. (**C**) High‐quality spectrum obtained from a properly processed sample, identified as *Mycobacterium avium* with a high‐confidence score. (**D**) High‐quality spectrum obtained from a properly processed sample, identified as *M. tuberculosis_*complex with a high‐confidence score.

In addition to the ID score, the software provides the corresponding mass‐to‐charge (m/z) values and signal intensities that define the spectral protein profile of each isolate. Visual inspection of peak distribution and intensity, and overall spectral resolution, is particularly useful when investigating low‐confidence scores or failed identifications, as poor‐quality spectra may result from insufficient biomass, incomplete cell wall disruption, carryover of culture medium, inadequate extraction, or target plate preparation issues. These outputs can be used to assess spectrum quality, compare technical replicates, and examine concordance with the reference library, as illustrated in Figure 2.

When replicate spots from the same isolate yield consistent high‐confidence scores, this supports the reproducibility of the extraction and acquisition procedure. Conversely, discrepant, or low‐confidence results should prompt review of the critical steps in biomass collection, inactivation, washing, bead‐beating, solvent extraction, and target spotting before repeating the analysis.

### Critical Parameters

Complete thermal inactivation is essential to ensure laboratory biosafety. The use of fresh cultures and careful collection of bacterial biomass are important to avoid the transfer of culture medium fragments, which may interfere with subsequent analysis. Cultures must be pure, as contamination prevents correct identification. Use newly prepared reagents and carry out sample identification, preferably on the same day as extraction.

When immediate analysis is not feasible or when access to the MALDI‐TOF instrument is limited, samples may be prepared up to Basic Protocol, step 13 (addition of acetonitrile) and stored at −20°C prior to analysis. In our experience, samples stored under these conditions for more than two weeks still yielded high‐quality spectra and reliable identification upon re‐analysis. Before use, samples should be thawed at room temperature, briefly mixed, and processed according to the subsequent steps of the protocol.

### Troubleshooting

See Table 2 for common problems encountered when performing this protocol and suggested solutions.

**Table 2:** Troubleshooting Guide for MALDI‐TOF MS–Based Identification of Mycobacteria

Problem	Possible cause	Solution
No peaks found	Insufficient biomass	Collect more biomass for the extraction
	Inadequate matrix	Prepare the matrix again
No identification possible	Old culture	Prepare a new culture
	Contaminated culture	Repeat the extraction using a pure culture
	Presence of culture medium in the sample	Repeat the extraction and avoid scraping the culture medium
	Microorganism not available in the library	Use a different library for comparison (e.g., MicrobeNet) or verify using another method (e.g., DNA sequencing)

### Statistical Analysis

Statistical analyses were performed using Statistical Software version 23.4.9 (MedCalc Software, Belgium). Each strain was analyzed in three independent experiments, and the score obtained in each experiment was used for statistical analysis.

Descriptive statistics (mean, median, variance, and standard deviation) were calculated. Variability between experiments was assessed using the coefficient of variation (*CV*), while reproducibility was evaluated using the intraclass correlation coefficient (*ICC*). The *CV* was 6.18%, and the *ICC* for average measures was 0.8235 (95% *CI*: 0.6848 to 0.9071), indicating good reliability of the results.

### Understanding Results

The efficiency of the protocol was evaluated through standardization using a set of reference mycobacterial strains (Table 3). The analysis of the protein extraction method in 33 reference strains for MALDI‐TOF MS identification demonstrated 100% concordance among the results obtained in the three independent rounds of analysis. All strains were correctly identified in all rounds performed (99/99 identifications), resulting in an overall agreement of 100%. For the strain *Mycobacterium austroafricanum*, identification as *Mycobacterium vanbaalenii* was observed in two rounds; however, these species are considered taxonomic synonyms according to the List of Prokaryotic Names with Standing in Nomenclature (2026).

**Table 3:** MALDI‐TOF MS Identification Results of Reference Mycobacterial Strains After Protein Extraction Using the Standardized Protocol*^a^*

Reference strain	MALDI‐TOF MS classification	1^st^ round result	Score	2^nd^ round result	Score	3^rd^ round result	Score
*M. abscessus* ATCC 19977	*M. abscessus*	*M. abscessus*	2.264	*M. abscessus*	2.205	*M. abscessus*	2.008
*M. aichiense* ATCC 27280	*M. aichiense*	*M. aichiense*	1.873	*M. aichiense*	1.914	*M. aichiense*	1.873
*M. austroafricanum* ATCC 33464	*M. austroafricanum/vanbaalenii*	*M. austroafricanum*	2.327	*M. vanbaalenii*	1.935	*M. vanbaalenii*	2.188
*M. avium* ATCC 25291	*M. avium*	*M. avium*	2.306	*M. avium*	2.167	*M. avium*	2.220
*M. bolleti* CCUG 50184	*M. abscessus*	*M. abscessus*	2.066	*M. abscessus*	1.881	*M. abscessus*	1.858
*M. bovis* ATCC 19274 BCG Moreau	*M. tuberculosis_complex*	*M. tuberculosis_complex*	2.055	*M. tuberculosis_complex*	2.253	*M. tuberculosis_complex*	2.226
*M. chelonae* ATCC 35752	*M. chelonae*	*M. chelonae*	2.085	*M. chelonae*	1.956	*M. chelonae*	2.200
*M. chubuense* ATCC 27278	*M. chubuense*	*M. chubuense*	1.841	*M. chubuense*	1.822	*M. chubuense*	1.961
*M. diernhoferi* ATCC 19340	*M. diernhoferi*	*M. diernhoferi*	2.055	*M. diernhoferi*	2.065	*M. diernhoferi*	1.973
*M. duvalli* ATCC 43910	*M. duvalii*	*M. duvalii*	2.095	*M. duvalii*	2.031	*M. duvalii*	1.928
*M. fortuitum* ATCC 6841	*M. fortuitum*	*M. fortuitum*	2.206	*M. fortuitum*	2.443	*M. fortuitum*	2.326
*M. intracellulare* ATCC 13950	*M. chimaera_intracellulare_group*	*M. chimaera_intracellulare_group*	2.198	*M. chimaera_intracellulare_group*	1.987	*M. chimaera_intracellulare_group*	2.067
*M. kansasii* Bostrumpi	*M. kansasii*	*M. kansasii*	2.143	*M. kansasii*	2.036	*M. kansasii*	2.028
*M. kansasii* Osaka	*M. kansasii*	*M. kansasii*	2.296	*M. kansasii*	2.241	*M. kansasii*	2.015
*M. massiliense* CCUG 48898	*M. abscessus*	*M. abscessus*	2.097	*M. abscessus*	2.191	*M. abscessus*	2.082
*M. moriokaense* ATCC 43059	*M. moriokaense*	*M. moriokaense*	1.820	*M. moriokaense*	1.890	*M. moriokaense*	1.867
*M. nonchromogenicum* ATCC 19530	*M. nonchromogenicum*	*M. nonchromogenicum*	2.096	*M. nonchromogenicum*	2.092	*M. nonchromogenicum*	2.208
*M. obuense* ATCC 27023	*M. obuense*	*M. obuense*	2.185	*M. obuense*	2.327	*M. obuense*	2.278
*M. parafortuitum* 2 ATCC 19686	*M. parafortuitum*	*M. parafortuitum*	2.049	*M. parafortuitum*	2.110	*M. parafortuitum*	2.245
*M. phlei* ATCC 11758	*M. phlei*	*M. phlei*	2.115	*M. phlei*	2.135	*M. phlei*	2.136
*M. porcinum* ATCC 33776	*M. porcinum*	*M. porcinum*	2.233	*M. porcinum*	2.261	*M. porcinum*	2.269
*M. pulveris* ATCC 35154	*M. pulveris*	*M. pulveris*	1.934	*M. pulveris*	1.927	*M. pulveris*	2.196
*M. rhodesiae* ATCC 27024	*M. rhodesiae*	*M. rhodesiae*	2.394	*M. rhodesiae*	2.192	*M. rhodesiae*	2.288
*M. smegmatis* ATCC 14468	*M. smegmatis*	*M. smegmatis*	2.043	*M. smegmatis*	2.047	*M. smegmatis*	1.906
*M. smegmatis* MC2155 ATCC 700084	*M. smegmatis*	*M. smegmatis*	1.902	*M. smegmatis*	2.094	*M. smegmatis*	2.129
*M. thermoresistibile* ATCC 19527	*M. thermoresistibile*	*M. thermoresistibile*	2.059	*M. thermoresistibile*	2.124	*M. thermoresistibile*	2.087
*M. triviale* ATCC 23292	*M. triviale*	*M. triviale*	2.064	*M. triviale*	1.886	*M. triviale*	2.049
*M. tuberculosis* H37Ra	*M. tuberculosis_complex*	*M. tuberculosis_complex*	2.093	*M. tuberculosis_complex*	2.235	*M. tuberculosis_complex*	2.137
*M. vaccae* ATCC 15483	*M. vaccae*	*M. vaccae*	2.232	*M. vaccae*	2.339	*M. vaccae*	2.303
*M. szulgai* ATCC 35799	*M. szulgai*	*M. szulgai*	1.891	*M. szulgai*	1.871	*M. szulgai*	1.968
*M. peregrinum* ATCC 700686	*M. peregrinum*	*M. peregrinum*	2.197	*M. peregrinum*	2.046	*M. peregrinum*	2.042
*M. immunogenum* ATCC 700505	*M. immunogenum*	*M. immunogenum*	1.831	*M. immunogenum*	1.828	*M. immunogenum*	1.950
*M. franklinii* DSM 45524	*M. franklinii*	*M. franklinii*	1.930	*M. franklinii*	2.082	*M. franklinii*	2.041

^*a*^ The table shows the identifications obtained in three independent rounds of analysis (1st, 2nd, and 3rd round), as well as the corresponding score values. The classification corresponds to the expected identification by MALDI‐TOF MS. Score values were interpreted according to the MBT BioTyper system criteria, in which scores ≥1.80 indicate high‐confidence identification.

In addition, some strains could only be identified at the complex level, as in the case of *M. tuberculosis* and *Mycobacterium bovis* (belonging to the MTBC) and *Mycobacterium intracellulare* (*Mycobacterium chimaera_intracellulare_group*). Species belonging to the *Mycobacterium abscessus* complex (MABC), such as *Mycobacterium bolletii* and *Mycobacterium massiliense*, were identified as *Mycobacterium abscessus*.

**Figure 3:**
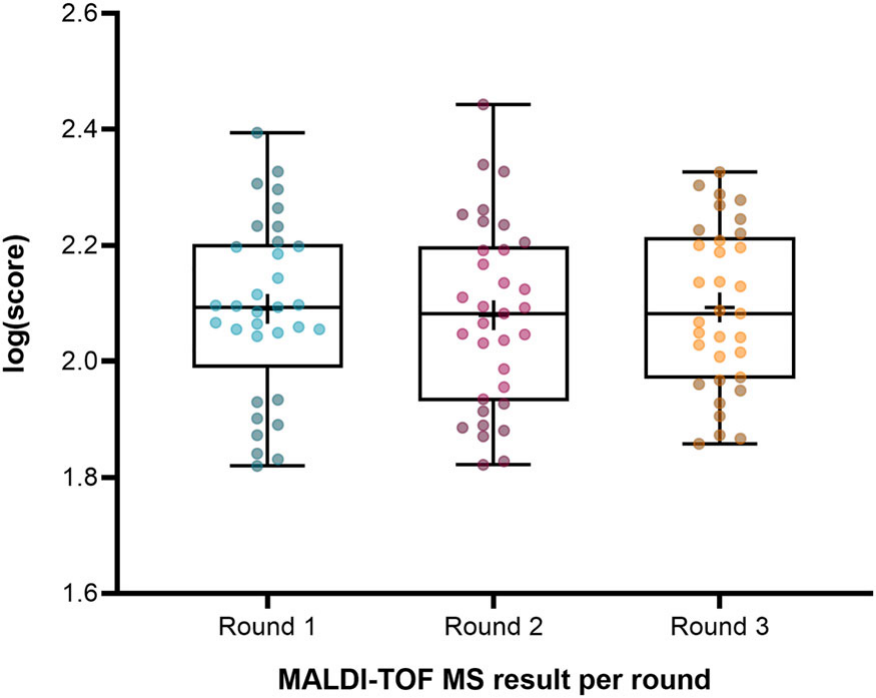
Distribution of MALDI‐TOF MS identification scores across three independent analysis rounds. The boxplot illustrates the distribution of score values obtained for each round, each point represents the score obtained for an individual strain. Boxes represent the interquartile range (*IQR*), the horizontal line indicates the median, and whiskers represent the minimum and maximum values. All scores were interpreted according to the Bruker MBT BioTyper criteria, where values ≥1.80 indicate high‐confidence identification.

This classification by MALDI‐TOF MS reflects the high spectral similarity among species within these complexes, a limiting factor for the technique in discriminating closely related species. Despite this, all identifications obtained showed score values ≥1.80, corresponding to high‐confidence identification according to the Bruker MBT BioTyper system criteria, as shown in Figure 3.

#### MALDI‐TOF MS quality control

Internal quality control for MALDI‐TOF MS was carried out using the Bacterial Test Standard (BTS, Bruker Daltonics), which consists of an *Escherichia coli* protein extract for instrument calibration.

### Time Considerations

The active time required for Basic Protocol is ∼150 min, including the steps of inactivation, washing, protein extraction, and sample application. Analysis on the MALDI‐TOF Sirius Biotyper takes ∼20 min to process a full 96‐spot target plate. The total processing time may vary depending on the number of samples being processed and the number of operators involved. For optimal workflow control and reproducibility, we recommend that no more than 6 samples be processed simultaneously per operator.

### Author Contributions

**Lucas Evangelista Marques**: Data curation; formal analysis; investigation; methodology; writing—original draft; writing—review and editing. **Stephanie Chrystine Balestro Mota**: Data curation; methodology; formal analysis; writing—review and editing. **Christoffel Opperman**: Data curation; formal analysis; methodology; writing—review and editing. **Robin Mark Warren**: Formal analysis; supervision; writing—review and editing. **Emilyn Costa Conceição**: Data curation; formal analysis; methodology; writing—original draft; writing—review and editing. **Cristina Viana‐Niero**: Conceptualization; data curation; formal analysis; funding acquisition; investigation; methodology; project administration; resources; supervision; validation; visualization; writing—original draft; writing—review and editing.

### Conflict of Interest

The authors declare no conflict of interest.

## Data Availability

The data, tools, and materials (or their source) that support the protocol are available from the corresponding author upon reasonable request.

## References

[cpz170425-bib-0001] Albay, A. , Hoşbul, T. , Uçarman, S. N. , Özcan, H. , Tekin, K. , & Arslantürk, A. (2022). Tüberküloz Dışı Mikobakterilerin Tanımlanmasında Üç Farklı Protein Ekstraksiyon Protokolü ve Matriks Destekli Lazer Desorpsiyon İyonizasyonUçuş Zamanlı Kütle Spektrometresi Etkinliğinin Araştırılması. Mikrobiyoloji Bulteni, 56(2), 206–217. 10.5578/mb.20229802 35477225

[cpz170425-bib-0002] Alcaide, F. , Amlerová, J. , Bou, G. , Ceyssens, P. J. , Coll, P. , Corcoran, D. , Fangous, M.‐S. , González‐Álvarez, I. , Gorton, R. , Greub, G. , Hery‐Arnaud, G. , hábak, J. , Ingebretsen, A. , Lucey, B. , Marekoviċ, I. , Mediavilla‐Gradolph, C. , Monté, M. R. , O'Connor, J. , O'Mahony, J. , … Rodriguez‐Sanchez, B. (2018). How to: Identify non‐tuberculous Mycobacterium species using MALDI‐TOF mass spectrometry. Clinical Microbiology and Infection, 24(6), 599–603. 10.1016/j.cmi.2017.11.012 29174730

[cpz170425-bib-0003] Bacanelli, G. , Araujo, F. R. , & Verbisck, N. V. (2023). Improved MALDI‐TOF MS Identification of Mycobacterium tuberculosis by Use of an Enhanced Cell Disruption Protocol. Microorganisms, 11(7), 1692. 10.3390/microorganisms11071692 37512865 PMC10386467

[cpz170425-bib-0004] Bruker Daltonics . (2014). Mycobacteria Extraction (MycoEX) Method: Standard Operating Procedure (pp. 1–4). Bruker Daltonics GmbH.

[cpz170425-bib-0005] Bruker Daltonics . (2026). *MBT HT Mycobacteria Module: MALDI Biotyper – Confident mycobacteria identification*. https://www.bruker.com/en/products‐and‐solutions/microbiology‐and‐diagnostics/microbial‐identification/mbt‐and‐mycobacteria‐identification.html

[cpz170425-bib-0006] Calderaro, A. , & Chezzi, C. (2024). MALDI‐TOF MS: A reliable tool in the real life of the clinical microbiology laboratory. Microorganisms, 12(2), 322. 10.3390/microorganisms12020322 38399726 PMC10892259

[cpz170425-bib-0007] Chikamatsu, K. , Aono, A. , Yamada, H. , Sugamoto, T. , Kato, T. , Kazumi, Y. , Tamai, K. , Yanagisawa, H. , & Mitarai, S. (2014). Comparative evaluation of three immunochromatographic identification tests for culture confirmation of Mycobacterium tuberculosis complex. BMC Infectious Diseases, 14(1), 54. 10.1186/1471-2334-14-54 24484470 PMC3916065

[cpz170425-bib-0008] Daffé, M. , & Marrakchi, H. (2019). Unraveling the structure of the mycobacterial envelope. Microbiology Spectrum, 7(4). 10.1128/microbiolspec.GPP3-0027-2018 PMC10957186 31267927

[cpz170425-bib-0009] El Khéchine, A. , Couderc, C. , Flaudrops, C. , Raoult, D. , & Drancourt, M. (2011). Matrix‐assisted laser desorption/ionization time‐of‐flight mass spectrometry identification of mycobacteria in routine clinical practice. PLoS ONE, 6(9), e24720. 10.1371/journal.pone.0024720 21935444 PMC3172293

[cpz170425-bib-0010] Forbes, B. A. , Hall, G. S. , Miller, M. B. , Novak, S. M. , Rowlinson, M.‐C. , Salfinger, M. , Somoskövi, A. , Warshauer, D. M. , & Wilson, M. L. (2018). Practical guidance for clinical microbiology laboratories: Mycobacteria. Clinical Microbiology Reviews, 31(2), e00038–17. 10.1128/CMR.00038-17 29386234 PMC5967691

[cpz170425-bib-0011] Gordillo‐Marroquín, C. , Sánchez‐Pérez, H. J. , Gómez‐Velasco, A. , Martín, M. , Guillén‐Navarro, K. , Vázquez‐Marcelín, J. , Gómez‐Bustamante, A. , Jonapá‐Gómez, L. , & Alocilja, E. C. (2022). Tween 80 improves the acid‐fast bacilli quantification in the magnetic Nanoparticle‐Based Colorimetric Biosensing Assay (NCBA). Biosensors, 12(1), 29. 10.3390/bios12010029 35049656 PMC8773761

[cpz170425-bib-0012] Huang, T.‐S. , Lee, C.‐C. , Tu, H.‐Z. , & Lee, S. S.‐J. (2018). Rapid identification of mycobacteria from positive MGIT broths of primary cultures by MALDI‐TOF mass spectrometry. PLOS ONE, 13(2), e0192291. 10.1371/journal.pone.0192291 29394275 PMC5796708

[cpz170425-bib-0013] Julián, E. , Cama, M. , Martínez, P. , & Luquin, M. (2001). An ELISA for five glycolipids from the cell wall of Mycobacterium tuberculosis. Journal of Immunological Methods, 251(1–2), 21–30. 10.1016/S0022-1759(01)00313-1 11292478

[cpz170425-bib-0014] List of Prokaryotic names with Standing in Nomenclature . (2026). *Genus Mycobacterium*. Online. https://lpsn.dsmz.de/genus/mycobacterium

[cpz170425-bib-0015] Lopes, M. , Batista, M. , Garcia, T. , Alves, H. , Boaventura, L. , Pontes, C. , & Rodrigues, F. (2023). Non‐tuberculous mycobacteria: Clinical and laboratory characterisation (2009 and 2019). Epidemiology and Infection, 151, e8. 10.1017/S0950268822000899 PMC9990399 36503567

[cpz170425-bib-0016] López‐Medrano, R. , Burgos‐Asurmendi, I. , & Rivero‐Lezcano, O. (2025). A simplified extraction method reduces the processing time for proteomic identification of nontuberculous mycobacteria. Enfermedades Infecciosas y Microbiologia Clinica (English Ed.), 43(7), 411–415. 10.1016/j.eimce.2025.03.016 40467411

[cpz170425-bib-0017] Lyamin, A. V. , Ereshchenko, A. A. , Gusyakova, O. A. , Yanchenko, A. V. , Kozlov, A. V. , & Khaliulin, A. V. (2023). Comparison of laboratory methods for identifying members of the family Mycobacteriaceae. International Journal of Mycobacteriology, 12(2), 129. 10.4103/ijmy.ijmy_68_23 37338472

[cpz170425-bib-0018] Machnik, K. , Smoliński, J. , & Paściak, M. (2024). Evaluation of protein extraction protocols for MALDI‐TOF Biotyper analysis of mycobacteria. Journal of Microbiological Methods, 227, 107052. 10.1016/j.mimet.2024.107052 39384072

[cpz170425-bib-0019] Martin, E. C. , Limousin, L. , Renaux, C. , Catherinot, E. , & Vasse, M. (2023). Evaluation of the mycobacteria MBT kit for identification of nontuberculous mycobacteria by MALDI‐TOF Biotyper (Bruker). Diagnostic Microbiology and Infectious Disease, 107(3), 116044. 10.1016/j.diagmicrobio.2023.116044 37657233

[cpz170425-bib-0020] Mather, C. A. , Rivera, S. F. , & Butler‐Wu, S. M. (2014). Comparison of the Bruker Biotyper and vitek MS matrix‐assisted laser desorption ionization–time of flight mass spectrometry systems for identification of mycobacteria using simplified protein extraction protocols. Journal of Clinical Microbiology, 52(1), 130–138. 10.1128/JCM.01996-13 24172150 PMC3911429

[cpz170425-bib-0021] Parish, T. , & Kumar, A. (Eds.). (2021). Mycobacteria Protocols (4th ed.). Springer US. 10.1007/978-1-0716-1460-0

[cpz170425-bib-0022] Pastrone, L. , Curtoni, A. , Criscione, G. , Scaiola, F. , Bottino, P. , Guarrasi, L. , Iannaccone, M. , Timke, M. , Costa, C. , & Cavallo, R. (2023). Evaluation of two different preparation protocols for MALDI‐TOF MS nontuberculous mycobacteria identification from liquid and solid media. Microorganisms, 11(1), 120. 10.3390/microorganisms11010120 36677412 PMC9866535

[cpz170425-bib-0023] Pignone, M. , Greth, K. M. , Cooper, J. , Emerson, D. , & Tang, J. (2006). Identification of mycobacteria by matrix‐assisted laser desorption ionization‐time‐of‐flight mass spectrometry. Journal of Clinical Microbiology, 44(6), 1963–1970. 10.1128/JCM.01959-05 16757585 PMC1489414

[cpz170425-bib-0024] Ramos, A. , Carvalho, T. , Ribeiro, M. , & Guimarães, J. T. (2016). Capilia^TM^ TB‐Neo assay: A new tool for rapid distinction between tuberculous and non‐tuberculous mycobacteria. The International Journal of Tuberculosis and Lung Disease, 20(6), 753–756. 10.5588/ijtld.15.0528 27155177

[cpz170425-bib-0025] Rodriguez‐Temporal, D. , Alcaide, F. , Mareković, I. , O'Connor, J. A. , Gorton, R. , van Ingen, J. , van den Bossche, A. N. , Héry‐Arnaud, G. , Beauruelle, C. , Orth‐Höller, D. , Palacios‐Gutiérrez, J.‐J. , Tudó, G. , Bou, G. , Ceyssens, P.‐J. , Garrigó, M. , González‐Martin, J. , Greub, G. , habak, J. , Ingebretsen, A. , … Rodríguez‐Sánchez, B. (2022). Multicentre study on the reproducibility of MALDI‐TOF MS for nontuberculous mycobacteria identification. Scientific Reports, 12(1), 1237. 10.1038/s41598-022-05315-7 35075208 PMC8786948

[cpz170425-bib-0026] Saleeb, P. G. , Drake, S. K. , Murray, P. R. , & Zelazny, A. M. (2011). Identification of mycobacteria in solid‐culture media by matrix‐assisted laser desorption ionization–time of flight mass spectrometry. Journal of Clinical Microbiology, 49(5), 1790–1794. 10.1128/JCM.02135-10 21411597 PMC3122647

[cpz170425-bib-0027] Toney, N. C. , Zhu, W. , Jensen, B. , Gartin, J. , Anderson, K. , Lonsway, D. , Karlsson, M. , & Rasheed, J. K. (2022). Evaluation of MALDI biotyper mycobacteria library for identification of nontuberculous mycobacteria. Journal of Clinical Microbiology, 60(9), e0021722. 10.1128/jcm.00217-22 35969171 PMC9491183

[cpz170425-bib-0028] Wang, C.‐H. , Putri, D. U. , Lee, J.‐C. , Liao, C.‐C. , Tsao, S. , Hsiao, A.‐L. , Wu, J.‐H. , Chen, X.‐W. , Lee, C.‐H. , & Tsai, I.‐L. (2021). Biosafety and proteome profiles of different heat inactivation methods for mycobacterium tuberculosis. Microbiology Spectrum, 9(3), e0071621. 10.1128/spectrum.00716-21 34937194 PMC8694153

[cpz170425-bib-0029] Weiss, Z. F. , & Basu, S. S. (2025). The mass spectrometry revolution in clinical microbiology Part 1. Clinics in Laboratory Medicine, 45(1), 1–13. 10.1016/j.cll.2024.10.011 39892929

[cpz170425-bib-0030] WHO, W. H. O. (2024). WHO consolidated guidelines on tuberculosis. Module 3: Diagnosis – rapid diagnostics for tuberculosis detection. In WHO (3rd ed.). 38527162

[cpz170425-bib-0032] Parish, T. , & Kumar, A. (Eds.). (2021). Mycobacteria Protocols, 4th ed. Springer. 10.1007/978-1-0716-1460-0

[cpz170425-bib-0035] https://microbenet.cdc.gov/

